# Children's Hospitals in East London

**Published:** 1893-04-29

**Authors:** 


### THE INSTITUTIONAL WORKSHOP.

### WITHIN THE HOSPITALS.

### CHILDREN'S HOSPITALS IN EAST LONDON

On a recent Saturday we went to Shadwell, and afterwards to Shoreditch, in order to inspect the East London Hospital, and the North-Eastern Hospital for Children. In order to reach the East London Hospital, which is situated in Glamis Road, Shadwell, we took the Metropolitan Railway to Shadwell Station. The hospital is situated about seven minutes' walk from the station, and is reached by passing down Sutton Street East to Cable Street, and thence to Glamis Road. Short as was the journey, it revealed much of the life and surroundings of the poor in this district. Sutton Street East was occupied by costers' barrows and constituted an open market. Although we arrived early in the afternoon there were many people about, and we could not fail to observe that the police have a good deal to do in this district.

The North-Eastern Hospital is situated in Shoreditch, and can be reached by the North London Railway. We traversed the distance between the East London and North-Eastern Hospitals in a cab, which enabled us to see a good deal of the neighbourhood and its inhabitants. The crowded condition of the streets, and the fact that the houses are for the most part in two storeys only, strike the visitor at once. These two-storey houses are infinitely preferable to the tenement houses to be met with in many parts of London, and in the Liberties of Westminster, where it is not uncommon to find a whole family crowded into one room. Although the districts of Shadwell and Shoreditch are mainly inhabited by poor people, they abound in small houses of two storeys, an arrangement which tends to lessen the evils of over-crowding, as it enables the majority at any rate to occupy a small house to themselves. This fact is often overlooked, although it has important bearings upon the morals and character of the people. Judging by the type of children to be found in the East London Hospital, and in the North-Eastern Hospital respectively, we should say that the poor are relatively of a higher type and greater intelligence in Shoreditch than they are in Shadwell. Be this as it may, we would urge all who have leisure to give up an afternoon, or afternoons, to enable them to visit these two institutions, and so to bring themselves into touch with certain aspects of London life, which cannot fail to interest them, and which, we hope, may induce them to take a warm and continued interest in the well-being of two children's hospitals situated faraway from the haunts of the well-to-do Londoner, which both need and deserve a much larger measure of pecuniary assistance than they have heretofore received from the public. There must be a large number of relatively idle people of means, who could brighten their own lives and immeasurably brighten the lives of the suffering children and of those who devotedly attend them in these hospitals, would they but take the trouble, if they cannot bring themselves to pay a visit to these institutions, or at any rate to send a contribution of toys, say once a month, and a contribution of flowers at least once a week throughout the year to the matron of one or both of these hospitals. As it is at present, the public for the most part know little of, and apparently care less for the sick children who are so well cared for at Shadwell and Shoreditch. Thus it comes to pass, as we shall show when we describe each of these hospitals in detail, that those who have to tend the sick, that is to say, the nurses, have also to bear the burden of brightening the wards with flowers and pictures, because, did they fail to do so, the poor children would be left without either one or the other. Toys are much too few for the needs and well-being of the children in these two hospitals, except perhaps at Christmas time, when the contributions from the *Truth* Toy Fund bring a temporary ray of sunlight into the lives of the poor little sufferers who here find a home and a refuge in the hour of their sorest need.

---

